# Is the 15-minute city within reach? Evaluating walking and cycling accessibility to grocery stores in Vancouver

**DOI:** 10.1016/j.trip.2022.100602

**Published:** 2022-04-18

**Authors:** Kate Hosford, Jeneva Beairsto, Meghan Winters

**Affiliations:** Faculty of Health Sciences, Simon Fraser University, 8888 University Drive, Burnaby, BC V5A1S6, Canada

**Keywords:** Accessibility, 15-minute city, Walking, Cycling, Food environments

## Abstract

Leaders around the world have embraced the idea of a “15-minute city”. This urban planning concept proposes a city where residents can meet their essential needs within a short walking or cycling trip from their home. Local access to grocery stores is a necessary component for cities to achieve the 15-minute city. This study aims to evaluate local accessibility to grocery stores by walking and cycling in the City of Vancouver. We used a cu-mulative opportunity measure to count the number of grocery stores available within a 15-minute walk and cycle from people’s homes. To evaluate accessibility from the perspective of younger and older travellers, we considered different travel speeds. Our results show there is good accessibility to grocery stores when cycling, with less than 1% of the city’s population not having a grocery store within a 15-minute cycle. When assuming a walking speed of an older pedestrian, around one-fifth of the population did not have access to a grocery store in their local area. The neighbourhoods that did not have a store within a 15-minute walk had higher proportions of children, older adults, and visible minorities, and lower rates of employment and post-secondary education attainment. In seeking to improve accessibility via walking and cycling, cities should prioritize grocery store locations and investments in pedestrian and cycling infrastructure to underserved neighbourhoods and populations.

## Introduction

The 15-minute city proposes a city where all residents can meet their daily needs within a short walking or cycling trip from their home (Moreno et al., 2021). This concept builds on long established planning and development principles which encourage walkability, a diversity of destinations, and density, such as the Neighbourhood Unit (Perry, 1929) and New Urbanism (Congress for the New Urbanism, 2022). In recent years, these principles have been repopularized through the 15-minute city, an urban planning model where residents can fulfil six essential urban social functions in their local neighbourhood: living, working, commerce, healthcare, education, and entertainment (Moreno et al., 2021). While the ‘15 min’ threshold is specified in the framing of the concept, the authors emphasize that the 15-minute city is not meant to be rigid in terms of time, but rather is rooted in proximity-based planning. They state the time threshold can be tailored to the specific contexts of cities (Moreno et al., 2021).

The concept has garnered considerable interest from cities and international organizations, however, some question whether this concept translates from a European to a North American context (O’Sullivan & Bliss, 2020). Many European cities actively pursuing the 15-minute city, such as Paris and Barcelona, already have relatively good local access to destinations compared to North American cities. The simile used in a 2020 Bloomberg captures this difference: “Transforming [European cities] is rather like giving a supermodel a makeover. The challenge is far greater in the kinds of younger, sprawling cities found in North American or Australia, where cars remain the dominant form of transit” (O’Sullivan & Bliss, 2020).

In this paper, we explored whether the 15-minute city is within reach in a Canadian context by evaluating local accessibility to grocery stores by walking and cycling in the City of Vancouver. A secondary aim was to explore social equity in access to grocery stores by comparing the sociodemographic characteristics of the population who had access to at least 1 grocery store within a 15-minute walk compared to those who did not. We focused exclusively on grocery stores because this amenity is an essential component of the 15-minute city and a destination that almost all households need to access on a regular basis. We selected Vancouver as our case study because it has the highest population density of any Canadian city – making it one of the more likely candidates to achieve the 15-minute city. Vancouver is also already working towards its towards its own vision of the 15-minute city as part of its Climate Emergency Response. The City of Vancouver has set target that 90% of residents live within an easy walk or roll of their daily needs by 2030 (City of Vancouver, 2020a).

## Methods

We used the cumulative opportunity measure to count the number of grocery and produce stores available within a 15-minute walk and cycle from people’s homes. The cumulative opportunity measure counts the number of opportunities reached within a given travel time or cost threshold, which makes it well suited to evaluate the 15-minute city concept.

We considered different travel speeds in the analysis to compare accessibility from the perspective of younger and older travellers.

### Setting

The study area for this analysis is the City of Vancouver. With a population of approximately 630,000 and a land area of 115 km^2^, Vancouver has the highest population density of any Canadian city (Statistics Canada, 2016). Vancouver is also considered one of the most amenity dense cities in Canada (Bozikovic et al., 2020), and has a reasonably good road network for pedestrians and cyclists in the North American context. Approximately half of shopping trips in Vancouver are made by walking (39%) or cycling (10%) (City of Vancouver, 2020b).

### Data

#### Origins

We used dissemination block centroids to represent the origins for all residents in a dissemination block. Dissemination blocks are geographical areas equivalent to one city block. They are the smallest geographical unit provided by Statistics Canada for which population counts are available (Statistics Canada, 2018), and therefore provide the most accurate estimate of accessibility for individuals and households. We excluded all dissemination blocks with zero population (529 out of 4570). The 4041 remaining dissemination blocks have a mean population of 156 (range: 5–1690) and a mean size of 0.024 km^2^ (range: 0.001–1.28 km^2^).

#### Destinations

We aimed to include food stores that provided access to healthy food options, which we considered to be supermarkets, grocery stores, and produce stores. We excluded convenience stores. We acquired the locations of Vancouver’s supermarkets, grocery stores, and produce stores from the Business Licenses dataset in the City of Vancouver’s Open data catalogue (downloaded March 2021) (City of Vancouver, 2021). Since the City of Vancouver shares a land border with the City of Burnaby, we also acquired the locations of grocery and produce stores from the City of Burnaby’s Open data catalogue (downloaded October 2021). We filtered business licenses’ to include the store types as shown in Table 1. Hereafter, we refer to all store types included as grocery stores.

#### Travel impedance

Impedance between origins and destinations was operationalized by measuring travel time along the street network. We used Open-StreetMaps (OSM) data for the street network. Since the distance covered along the street network in 15-minutes will depend on the traveller’s speed, which may be impacted by age or topography, we used different values for walking and cycling speeds of younger and older travellers. For pedestrians, we used speeds provided in the Highway Capacity Manual: 4.8 km/hr for younger pedestrians (<65 years) and 3.6 km/hr for older pedestrians (≥ 65 years) (Transportation Research Board, 2016). For cyclists, we used the average speeds from a study that measured speeds of cyclists in different age groups: 16.2 km/hr for younger cyclists (<65 years) and 13.9 km/hr for older cyclists (≥ 65 years) (Schleinitz et al., 2017).

#### Analysis

To generate travel time matrices between dissemination block centroids and grocery stores, we used the R package R5R (Pereira et al., 2021). The travel time matrix function in R5R calculates origin-destination travel times using the street network from OSM and allows for specifications of mode, speed, max trip duration, and Level of Traffic Stress (LTS).

The R5 routing engine makes use of OSM’s tags to determine what streets are suitable for walking and cycling. Tags are used to describe specific features in OSM. For example, the R5 routing engine removes all road segments with the tag “highway = motorway” when determining routes for pedestrians and cyclists. For cycling, a maximum LTS can be specified to further filter the network to only include routes where most cyclists would feel comfortable cycling. LTS is a rating system for classifying streets and intersections based on tolerance for traffic stress (Mekuria et al., 2012). The scoring ranges from 1 to 4, where LTS 1 represents the lowest stress roads that children could tolerate (e.g., roads with protected bike lanes) and LTS 4 represents the highest stress roads that only ‘strong and fearless’ cyclists would tolerate (e.g., higher speed, multilane roads with no bike lanes). To calculate the cycling travel time matrices, we specified a maximum LTS of 2 (e.g., cyclists are either physically separated from traffic or cycling on low volume and low speed streets), which is the level of traffic stress that is tolerated by most adults (Mekuria et al., 2012).

We generated four travel time matrices for the following scenarios: (1) walking: younger traveller (speed 4.8 km/hr); (2) walking: older traveller (3.6 km/hr); (3) cycling: younger traveller (16.2 km/hr); and (4) cycling: older traveller (13.9 km/hr). The travel time matrices were used to count the number of grocery stores within a 15-minute travel time of each dissemination block in the four scenarios. We calculated descriptive statistics to summarize the number of grocery stores within 15-minutes.

To examine social equity in access to grocery stores, we compared the average sociodemographic characteristics between two groups: those who did not have a grocery store within a 15-minute walk to those who had at least one store within a 15-minute walk (assuming a walking speed of 3.6 km/hr). Since population-level sociodemographic characteristics are not available at the dissemination block level, we calculated the average number of grocery stores accessible in each dissemination area (DA). For both groups (DAs with ‘no access’, and DAs with ‘access to 1 or more stores’), we calculated the average proportion for seven sociodemographic variables: % aged 0–14 years, % aged 65 and over, employment rate, % with post-secondary education, % low income, % visible minority, % of recent immigrants. We used the after-tax low-income measure calculated by Statistics Canada (Statistics Canada, 2017). Visible minority is defined by Statistics Canada as “persons, other than Aboriginal peoples, who are non-Caucasian in race or non-white in colour”(Statistics Canada, 2021).

## Results

**Fig. 1:**
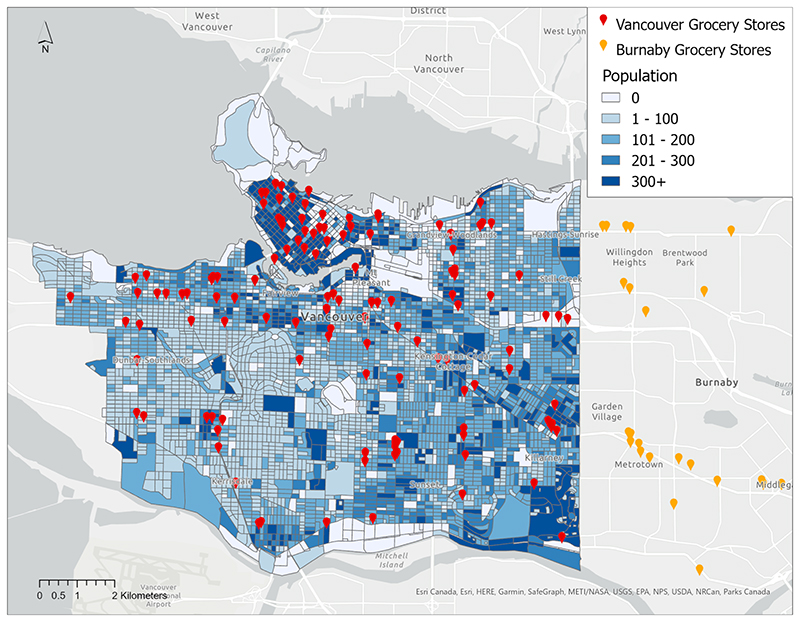
Location of grocery stores in Vancouver and Burnaby, and population distribution in Vancouver.

A total of 169 grocery stores were identified in the study area (n = 131 in Vancouver and n = 38 in Burnaby). Fig. 1 maps the location of the grocery stores and the population distribution across Vancouver. Grocery stores tend to be located in areas with higher populations, and the highest concentration is located in the downtown core, the most densely populated area of the city. The south-west region of Vancouver, an area with lower population density, has the lowest concentration of grocery stores.

**Fig. 2:**
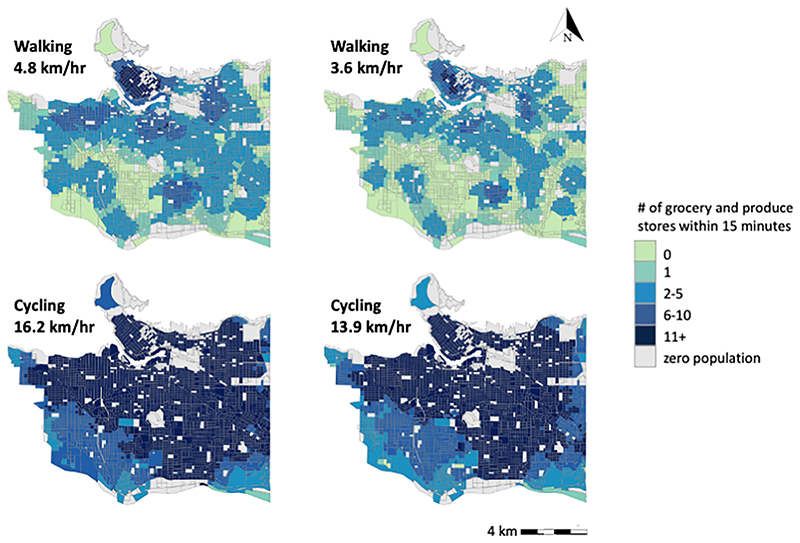
Accessibility to grocery stores by walking and cycling at two travel speeds, Vancouver, BC.

The majority of dissemination blocks in the City of Vancouver were within a 15-minute walk of at least one grocery store and almost all dissemination blocks were within a 15-minute cycle (Fig. 2 and Table 2). However, there is a large proportion of the population that only had access to 1 or 2 grocery or produce stores within a 15-minute walk. As expected, those with faster travel speeds have greater accessibility to grocery stores.

### Accessibility by walking

The dissemination blocks with the greatest accessibility to grocery stores by walking are in the downtown core and surrounding neigh-bourhoods. Dissemination blocks with poor accessibility to grocery stores were concentrated in the south area of city.

Assuming a walk speed of a younger pedestrian, dissemination blocks had access to 4 grocery stores within 15 minutes, on average. Only 11% of dissemination blocks did not have a grocery store within 15 minutes, which corresponds to roughly 9% of the city’s population. An additional quarter of the city’s population only had one or two stores within 15 minutes at a walking speed of a younger pedestrian.

Accessibility to grocery stores by walking was reduced considerably when a walking speed of an older pedestrian was assumed. Dissemination blocks had an average of 2 grocery stores within 15 minutes. Over a quarter of dissemination blocks (27%) had no grocery store within 15 minutes, which corresponds to about one fifth (21%) of the city’s population. An additional third (34%) of the population only had one or two stores within 15 minutes at a walking speed of an older pedestrian.

### Accessibility by cycling

There is good accessibility by cycling to grocery stores across the city. The majority of dissemination blocks had access to more than 10 grocery stores within a 15-minute cycle. Dissemination blocks in the corners of the city – particularly the south-west neighbourhoods – have lower accessibility.

Assuming a cycling speed of a younger cyclist, dissemination blocks had an average of 20 grocery stores within a 15-minute cycle. The majority of dissemination blocks (80%) had access to 11 or more stores, which corresponds to 85% of the city’s population. There are no dissemination blocks without access to a grocery store within 15 minutes.

For cycling speeds of older travellers, dissemination blocks had an average of 16 stores within a 15-minute cycle. Nearly three-quarters of the population (72%) had access to 11 or more grocery stores, and only 5 dissemination blocks did not have access to any grocery store within 15 minutes.

### Socio-demographic analysis

Table 3 compares the socio-demographic characteristics of dissemination areas with no grocery store within a 15-minute walk to those that have at least one store. Dissemination areas without access to grocery stores have, on average, higher proportions of children, older adults, and visible minorities. Dissemination areas with access to at least one grocery store have on average, greater proportions of people who are employed and have post-secondary education. There are no substantial differences in access by income or immigration status.

## Discussion

Our findings suggest that the 15-minute city is within reach in the City of Vancouver in terms of access to grocery stores. There is particularly good accessibility by cycling, with over three-quarters of the population having over 10 grocery stores within a 15-minute cycle. This highlights the important role that cycling can play in achieving the 15-minute city in the North American context. In terms of accessibility by walking, most dissemination blocks had at least one grocery store within 15-minutes, however in many areas the options were limited to one or two stores. We found that neighbourhoods that did not have access to a grocery within 15-minutes had higher proportions of children, older adults, visible minorities, and had lower employment rates and levels of post-secondary attainment.

The findings from this study highlight the importance of considering different perspectives in working towards the 15-minute city. We considered travel speed since this affects the number of amenities available within a 15-minute walk or cycle. At a walk speed typical of adults younger than 65 years, only 9% of the population did not have a grocery store within a 15-minute walk. At a walk speed typical of adults older than 65 years, the proportion that did not have a grocery store within 15-minutes more than doubled to 21% of the population. Applying more conservative estimates of walk-speed in evaluating local access to amenities will ensure that the 15-minute city is accessible to more people. Another perspective that could be considered in a future analysis is accessibility by cycling from the perspective of children or parents travelling with children. We specified a maximum LTS of 2 for routing cyclists along the street network, which includes routes with a level of traffic stress that is tolerable by most adults. To consider accessibility from the perspective of children, a maximum LTS of 1 could be specified, which would include only the lowest traffic stress routes.

Future work should also study perceptions of accessibility to consider whether the local opportunities available are meeting the needs of residents in the area. The accessibility analysis in this paper provides a starting point for identifying areas where there are high and low levels of accessibility to grocery stores by walking and cycling. A complementary body of work assesses people perceptions of their local neighbourhood, including the perceived safety and comfort of walking and cycling environments, the amount of time that people are willing to walk or cycle to amenities, and whether the local opportunities serve resident’s needs (Tiznado-Aitken et al., 2020; van Wee, 2016). We can envision future mixed methods research that integrates accessibility analyses with findings from qualitative interviews that investigates resident’s experiences of their local environment.

A second area for future research to explore is the role of online shopping in the 15-minute city. Widespread use of digital technology is changing the way we access services and opportunities (van Wee, 2016). According to data cited in a recently published study, the pandemic increased the use of online grocery services in Canada from prepandemic levels, however the vast majority of grocery trips were still in-person several months into the pandemic (86% of all grocery trips) (Goddard, 2021). Only 7% of Canadians surveyed felt that grocery shopping online was easier (Goddard, 2021). This suggests that physical access to a grocery store remains an important amenity, at least in the near term.

We used a cumulative opportunity measure to quantify the number of grocery stores within a 15-minute threshold. The advantage of this measure is that it provides an output that is absolute, easy to interpret, and comparable across modes and contexts (Levine et al., 2019). On the other hand, it does not account for the quality of destinations, nor the fact that distances that are further away may be less desirable than those that are closer (Levine et al., 2019). This can be addressed through gravity-based measures of accessibility, which weight destinations as a function of travel time, distance, or quality (Levine et al., 2019). Destinations that are closer are typically weighted higher; and destinations that are larger are weighted higher, often measured by the number of jobs or retail floor area ratio. As such, the closer the opportunity and the larger the opportunity, the more it contributes to accessibility. Although gravity-based measures improve upon the limitations of the cumulative-opportunity, the outputs are less intuitive to interpret because it is dimensionless (Levine et al., 2019). A recent paper that compared the cumulative opportunity measure to gravity-based measures found strong correlation between the two measures, and recommended the cumulative opportunity measure for its ease of communication and operationalization (Palacios & El-geneidy, 2022) We chose the cumulative opportunity measure for these reasons, in addition to the fact that the output (# of grocery stores within 15 minutes) is well suited to evaluating the 15-minute city concept.

There are a few limitations relating to the data used in this analysis. The grocery stores identified from the business licenses dataset may not capture all stores. A validation study of the 2015 Business Licenses data in the City of Vancouver found that the dataset undercounted the number of grocery stores compared to ground-truthed observational data (Daepp & Black, 2017). However, the authors concluded that the Business Licenses data was the best available dataset among municipal and commercial datasets in Vancouver. For the street network, we relied on OSM data. OSM is openly available, provides a network for all modes, and is continually updated; however, it is important to acknowledge that there is some degree of error in OSM data. Since OSM is built through crowdsourced volunteered geographic information, the quality of data will depend on those who are contributing to the map. A recent study found relatively good agreement between the overall length of bicycle infrastructure between OSM and municipal data, but a lack of consistency in the labelling of infrastructure (Ferster et al., 2020). These in-consistencies have implications for the LTS assigned to the street network, and therefore, also affect routing for cyclists according to LTS. In addition, small connections in the pedestrian network which increase accessibility, such as stairs and cut throughs, may be missing in OSM data. Finally, future studies on the 15-minute city may wish to evaluate access to a diversity of destinations beyond groceries stores, such as parks, recreation centres, restaurants, schools, health care, and other essential services.

## Conclusion

The 15-minute city envisions a city where all residents can access their daily needs within a short walking or cycling trip. This study evaluated whether the 15-minute city was within reach for the City of Vancouver in terms of access to grocery stores. Overall, our findings showed that the 15-minute city is within reach in the Vancouver context. Four fifths (79%) of Vancouver’s population had access to a grocery store within a 15-minute walk or cycle, assuming a travel speed of an older traveller. We found there was some inequities in access to grocery stores, with lower access in areas that had higher proportions of children, older adults, visible minorities, and lower employment and post-secondary education attainment rates. To help guide the development of a 15-minute city that is inclusive of all ages and abilities we recommend that researchers and city planners use conservative estimates of walk speed and level of traffic stress for cycling, while prioritizing investments that ensure more equitable access across neighbourhoods and populations.

## Figures and Tables

**Table 1:** Description of data sources.

Data	Source	Selection
**Origins**		
Dissemination block centroids	Abacus Data Network, Statistics Canada (2016)	All non-zero population dissemination blocks
**Destinations**		
Vancouver grocery stores	City of Vancouver Open Data catalogue (2021)	Business types: Retail Dealer – Market Outlet* Retail Dealer – Grocery** Retail Dealer – Food (where business subtype is ‘Produce’)
Burnaby grocery stores	City of BurnabyOpen Data catalogue (2021)	Licence types: Retail Trader – Food 1–10 persons (where store name contains ‘produce’, ‘supermarket’, ‘nesters’, ‘choices’, ‘buy-low’, ‘kin’s’) Retail Trader – Food 11–50 Retail Trader – Food 51+
**Travel impedance**		
Street network	OpenStreetMaps (2021)	Walking: All streets Cycling: Streets with Level of Traffic Stress 1 and 2
Walking speed	The Highway Capacity Manual 6th Ed (2016)	3.6 km/hr (older pedestrian) 4.8 km/hr (younger pedestrian)
Cycling speed	Schleinitz et al. (2017)	16.2 km/hr (older cyclist) 13.9 km/hr (younger cyclist)

*includes stores that are licensed to sell food and have a total floor area greater than 4,645 square meters (e.g., Superstore, Costco). **includes stores that are smaller than market outlets but have two or more of a bakery, butcher, and delicatessen (e.g., Safeway).

**Table 2:** Accessibility to grocery stores in the City of Vancouver from the perspective of a younger and older traveller (n = 4041 dissemination blocks with a non-zero population).

		Traveller^†^ Younger	Older
Number of grocery/ produce stores within a 15-minute walk	Mean	3.8	2.2
	Standard deviation	3.2	2.3
	Min	0	0
	Max	20	14
	# DB with zero stores (% of total DB)	458 (11.3%)	1071 (26.6%)
	Population with zero stores (% of total pop)	54.016 (8.6%)	133,680 (21.2%)
Number of grocery stores within a 15-minute cycle*	Mean	19.8	15.7
	Standard deviation	9.2	7.4
	Min	1	0
	Max	48	40
	# DB with zero stores (% of total DB)	0 (0%)	5 (<1%)
	Population with zero stores (% of total pop)	0 (0%)	240 (<1%)

DB = dissemination block. *Travel time based on cycling along streets with a maximum LTS of 2. ^†^ Younger travellers are assumed to have a walk speed of 4.8 km/hr and cycle speed of 16.2 km/hr and older travellers are assumed to have a walk speed of 3.6 km/hr and cycle speed of 13.9 km/hr.

**Table 3:** Average sociodemographic characteristics of dissemination areas with no access to grocery stores and dissemination areas with access to 1 or more grocery stores within a 15-minute walk.

	Access to store within a 15-minute walk^†^		Overall
	No	Yes	
Dissemination areas, n (% of total)	209 (21%)	784 (79%)	993
Socio-demographic characteristics			
0–14 years (%)	13.3	11.2	11.6
65 years and over (%)	19.3	15.1	16.0
Employment rate	57.9	64.1	62.8
Post-secondary education (%)	56.1	63.2	61.7
Low income* (%)	17.1	18.8	18.4
Visible minority (%)	65.1	48.2	51.7
Recent immigrant (%)	5.6	6.0	5.9

*based on the after-tax Low-Income Measure from Statistics Canada.(Statistics Canada, 2017). ^†^ based on a walk speed of 3.6 km/hr.
